# Cost-effectiveness of problem-solving treatment in comparison with usual care for primary care patients with mental health problems: a randomized trial

**DOI:** 10.1186/1471-2296-13-98

**Published:** 2012-10-10

**Authors:** Judith E Bosmans, Bettine Schreuders, Harm WJ van Marwijk, Jan H Smit, Patricia van Oppen, Maurits W van Tulder

**Affiliations:** 1 Department of Health Sciences and EMGO Institute for Health and Care Research, Faculty of Earth and Life Sciences, VU University Amsterdam, Amsterdam, The Netherlands; 2 Department of General Practice and EMGO Institute for Health and Care Research, VU University Medical Center, Amsterdam, The Netherlands; 3 Department of Psychiatry and EMGO Institute for Health and Care Research, VU University Medical Center, Amsterdam, The Netherlands; 4 Department of Epidemiology & Biostatistics and EMGO Institute for Health and Care Research, VU University Medical Center, Amsterdam, The Netherlands

**Keywords:** Costs and cost analysis, Problem-solving treatment, Nurses, Depression, Anxiety, Primary health care

## Abstract

**Background:**

Mental health problems are common and are associated with increased disability and health care costs. Problem-Solving Treatment (PST) delivered to these patients by nurses in primary care might be efficient. The aim of this study was to evaluate the cost-effectiveness of PST by mental health nurses compared with usual care (UC) by the general practitioner for primary care patients with mental health problems.

**Methods:**

An economic evaluation from a societal perspective was performed alongside a randomized clinical trial. Patients with a positive General Health Questionnaire score (score ≥ 4) and who visited their general practitioner at least three times during the past 6 months were eligible. Outcome measures were improvement on the Hospital Anxiety and Depression Scale and QALYs based on the EQ-5D. Resource use was measured using a validated questionnaire. Missing cost and effect data were imputed using multiple imputation techniques. Bootstrapping was used to analyze costs and cost-effectiveness of PST compared with UC.

**Results:**

There were no statistically significant differences in clinical outcomes at 9 months. Mean total costs were €4795 in the PST group and €6857 in the UC group. Costs were not statistically significantly different between the two groups (95% CI -4698;359). The cost-effectiveness analysis showed that PST was cost-effective in comparison with UC. Sensitivity analyses confirmed these findings.

**Conclusions:**

PST delivered by nurses seems cost-effective in comparison with UC. However, these results should be interpreted with caution, since the difference in total costs was mainly caused by 3 outliers with extremely high indirect costs in the UC group.

**Trial registration:**

Nederlands Trial Register ISRCTN51021015

## Background

Mental health problems are prevalent, with depression and anxiety being most common [1-3]. The disability burden of mental health problems is enormous [3]. Moreover, mental health problems are associated with high health care costs and lost productivity costs, thus causing a huge economic burden as well [4-6].

Most patients with mental health problems receive primary care [7]. The perceived burden on general practitioners (GPs) when treating these patients is higher than when treating other patients [8]. Treatment mainly consists of psychotropic drugs [7,9], although these drugs have disadvantages such as side effects, dependency and poor compliance. Moreover, many patients prefer psychological treatments over psychotropic drugs and advice [10,11]. However, most GPs have little experience in delivering psychological interventions and there is little evidence on the effectiveness of psychological treatments delivered by GPs [12]. Furthermore, it is questionable whether busy GPs have time to provide psychological treatment to patients with mental health problems. Psychological treatments could be made more widely available, if other health care providers could provide these treatments in an integrated primary care setting.

Problem-Solving Treatment (PST) is a psychological treatment that has been shown to be effective for depression in primary care and that can be effectively delivered by nurses or GPs [13,14]. However, for unselected patients with mental health problems who constitute the bulk of the primary care caseload, evidence of the effectiveness of PST was less clear and PST was associated with significantly higher health care costs than usual GP care [15,16]. The aim of this study was to assess the cost-effectiveness of PST by mental health nurses compared with usual GP care for primary care patients with mental health problems in The Netherlands.

## Methods

This economic evaluation was conducted alongside a randomized controlled trial with nine month follow-up that was performed in 12 general practices in and around Amsterdam, The Netherlands, between November 2003 and May 2005. The Medical Ethical Committee of the University Medical Center in Amsterdam approved the study protocol. Block randomization was used to allocate patients to either PST provided by a mental health care nurse or usual care from the GP. All patients gave written informed consent before randomization. The methodological details of the trial and the three months effectiveness results are reported in detail elsewhere [17,18].

### Patient selection

Consecutive patients who visited their GP were invited to complete the General Health Questionnaire (GHQ-12) [19]. Patients were eligible for the trial if they had a score of 4 points or more on the GHQ-12 [15,20], and visited their GP three times or more in the previous six months. Patients had to be 18 years or older, able to speak Dutch, and willing to undergo brief psychological treatment. Exclusion criteria were the existence of potentially life-threatening somatic and mental disorders, the existence of somatic and mental disorders limiting the patient’s ability to participate or adhere to treatment, and mental health treatment during the previous year.

### Usual care

Treatment of mental health problems in the usual care (UC) group was not restricted in any way. Dutch GPs are encouraged to work according to guidelines issued by the Dutch College of GPs [21,22]. The depression guidelines recommend that treatment of depression primarily consists of education and coaching. Antidepressant treatment and/or referral for psychotherapy can be added, depending on the duration and severity of the depressive symptoms, the limitations in daily functioning, and the patient’s preference [22]. For anxiety, the guidelines recommend referral for psychotherapy and medication if indicated [21].

### PST

PST is a brief treatment focused on practical skill building, education, and managing depressive symptoms. The goal is to stimulate an active attitude towards everyday problems and, hereby, to achieve a reduction in mental health problems. Twelve nurses working at one of the mental healthcare organizations in Amsterdam were trained during two days by two researchers who developed PST for primary care, L. Mynors-Wallis and I. Davies. In the second part of the training, the nurses treated four patients closely supervised by a cognitive behavioral therapist (PvO). The supervisor was certified as supervisor by the National Association of Behavior Therapy and Cognitive Therapy in The Netherlands.

Patients were offered four to six PST sessions. The first session lasted a maximum of 60 minutes and following sessions a maximum of 30 minutes. Patients could visit their GP for UC if necessary.

### Clinical outcome measures

The participants received written questionnaires at baseline, and at three and nine months after baseline. The primary clinical outcome was severity of mental health symptoms measured using the Hospital Anxiety and Depression Scale (HADS) [23]. Lower scores on the HADS indicate less severe symptoms. Quality of life was measured using the EQ-5D [24]. The EQ-5D scores were used to calculate utilities using the Dutch tariff [25]. QALYs were calculated using linear interpolation between time points. Higher QALY scores indicate more improvement in quality of life.

### Cost measures

Cost data were collected from a societal perspective 3 times during the 9 months follow-up using the TiC-P questionnaire with a recall period of 3 months [26]. If available, Dutch guideline prices were used to value resource use [27,28]. Medication was valued using prices of the Royal Dutch Society for Pharmacy [29]. Costs of visits to complementary therapists were based on prices from the therapists themselves. Lost productivity costs were calculated according to the friction cost approach (friction period 154 days) using the mean age- and sex-specific income of the Dutch population [28]. According to the friction cost approach a sick employee is replaced after a certain amount of time (the friction period) after which there are no lost productivity costs anymore. Table 1 lists the cost categories included in the economic evaluation and the prices used. All costs were adjusted to the year 2012 using consumer price indices [30]. 

**Table 1:** Health care utilization over 9 months of patients with complete cost data

**Category**	**Price (€)**	**PST (n=56)**	**Usual care (n=65)**	**Difference**
Primary care				
General practitioner [no contacts]	23.53	4.1 (3.3)	4.8 (4.5)	−0.7 (−2.1 ; 0.8)
Physiotherapist [no contacts]	26.50	5.5 (10.3)	6.0 (11.0)	−0.5 (−4.4 ; 3.4)
Social worker [no contacts]	55.75	0.3 (1.9)	0.2 (0.8)	0.1 (−0.5 ; 0.5)
Psychologist [no contacts]	88.53	1.1 (5.0)	1.0 (2.7)	0.1 (−1.3 ; 1.6)
Secondary care				
Outpatient clinic [no contacts]	65.23	2.3 (3.4)	4.2 (10.2)	−1.9 (−4.6 ; 0.8)
Regional institute for mental welfare [no contacts]	144.44	0.3 (0.9)	0.5 (1.9)	−0.2 (−0.8 ; 0.3)
In-hospital psychiatrist [no contacts]	73.88 – 131.94*	0.1 (0.7)	0.2 (0.9)	−0.1 (−0.4 ; 0.2)
Day treatment [no half days]	104.28 – 186.21*	0 (0)	0 (0)	--
Hospital admission [no days]	291.21 – 554.45^†^	0.3 (1.1)	1.3 (5.8)	−1.0 (−2.4 ; 0.5)
Home care [no hours]	35.76	4.2 (16.0)	7.9 (29.4)	−3.7 (−12.4 ; 5.0)
Complementary therapists [no contacts]	26.79 – 143.28^‡^	1.4 (3.8)	1.8 (4.8)	−0.4 (−1.9 ; 1.2)
Occupational physician [no contacts]	24.75	0.4 (1.4)	0.8 (1.6)	−0.4 (−0.9 ; 0.2)
Medication [no deliveries]	depending on type and dose	7.0 (6.8)	7.4 (6.5)	−0.4 (−2.8 ; 2.0)
PST [no contacts]	56.87^§^	4.1 (2.1)	--	
Help from family/friends or paid help [no hours]	10.36	14.9 (32.8)	12.5 (34.2)	2.4 (−9.8 ; 14.5)
Absenteeism [no days]	depending on age and sexe	19.5 (54.8)	29.0 (62.4)	−9.5 (−30.8 ; 11.8)
Presenteeism [no days]	depending on age and sexe	3.8 (12.9)	7.2 (19.9)	−3.4 (−10.0 ; 2.7)

Presented are means (SD) and mean differences (95% CI).

PST = Problem-Solving Treatment.

* depending on type of hospital: academic, general or psychiatric hospital.

^†^ depending on type of hospital: academic, general, rehabilitation or psychiatric hospital.

^‡^ depending on type of therapist.

^§^ Total PST costs based on 6 sessions: €341.24 (training €45.08, treatment €244.24, housing €51.92).

### Analysis

It was estimated that 65 patients in each group would be needed to detect an effect size of 0.4 (2-sided α=0.05, β=0.20). Taking into account a drop-out rate of 20%, the aim was to include 160 patients into the trial.

The statistical analyses were performed according to the intention-to-treat principle. Multiple Imputation (MI) according to the Multivariate Imputation by Chained Equations (MICE) algorithm was done to impute missing cost and effect data with SPSS 17.0 for Windows (SPSS Inc., Chicago, IL, USA). Predictive mean matching was used to account for the skewness of the cost data and the fact that costs are bounded by zero. An imputation model was constructed that included variables that were related to missingness or predicted the outcome variable. By MI 5 imputed data sets were created, each of which was analyzed separately. The results of the 5 analyses were pooled using Rubin’s rules [31].

Linear regression was used to estimate differences in costs and effects. Costs generally have a highly skewed distribution. Therefore, bootstrapping with 5000 replications was used to estimate “approximate bootstrap confidence” (ABC) intervals around cost differences [32,33].

Incremental cost-effectiveness ratios (ICERs) were calculated by dividing the difference in total costs between PST and UC by the difference in clinical effects. Non-parametric bootstrapping was also used to estimate the uncertainty surrounding the incremental cost-effectiveness and cost-utility ratios (5000 replications). The bootstrapped cost-effect pairs were plotted on a cost-effectiveness plane [34] and used to estimate cost-effectiveness acceptability curves. Cost-effectiveness acceptability curves show the probability that the intervention is cost-effective in comparison with the control treatment for a range of ceiling ratios. The ceiling ratio is defined as the amount of money society is willing to pay to gain one unit of effect [35].

### Sensitivity analyses

Six sensitivity analyses were performed to assess the robustness of the results. In the first sensitivity analysis, statistical analysis was restricted to patients with complete follow-up data. Secondly, we redid the economic evaluation from a NHS perspective meaning that only direct costs were included in the analyses. In the third sensitivity analysis, lost productivity costs were calculated according to the human capital approach. This approach assumes that lost productivity costs are generated until an employee recovers and returns to work, or until the moment of death or retirement. In the fourth sensitivity analysis, training costs were excluded from the PST intervention costs. The fifth sensitivity analysis concerned a per protocol analysis in which only PST patients were included who completed 4 or more PST sessions. Finally, in the sixth sensitivity analysis the observed outliers with very high lost productivity costs were excluded from the analysis.

## Results

During the inclusion period, 2133 patients completed the GHQ-12 and 353 refused to participate. 622 patients had a score of 4 or more on the GHQ-12 and visited their GP three times or more in the past 6 months. 311 patients were unwilling to participate in the trial and 136 did not meet the inclusion criteria, leaving 175 patients to be included in the trial of whom 88 were randomized to the PST group and 87 to the UC group.

At baseline, utility was significantly lower in UC patients than in PST patients meaning that the health status of UC patients was lower than of PST patients. All analyses concerning QALYs were corrected for this baseline difference. There were no other significant differences in clinical and demographic characteristics between the treatment groups at baseline (Table 2).

**Table 2:** Baseline characteristics

	**PST (n=88)**	**Usual care (n=87)**
Mean (SD) age (years)	52 (16)	53 (16)
Female	68 (77%)	57 (66%)
Married/cohabiting	34 (39%)	33 (38%)
Education level		
Low	17 (19%)	14 (16%)
Medium	22 (25%)	23 (26%)
High	31 (35%)	32 (37%)
Unknown	18 (21%)	18 (21%)
Born in The Netherlands	62 (71%)	60 (69%)
Mean EuroQol utility (SD)	0.73 (0.18)	0.62 (0.28)
Mean HADS score (SD)	15.4 (7.5)	16.9 (7.1)

Presented are numbers (%) unless stated otherwise.

HADS = Hospital Anxiety Depression Scale.

Complete cost data were available for 56 (64%) of the PST patients and 65 (75%) of the UC patients. There were no significant differences in baseline characteristics between patients with and without complete cost data.

### Clinical effects

Clinical effects are presented in Table 3. After 9 months the multiply imputed pooled improvement in HADS score was −3.1 (SE 1.3) in PST patients and −2.9 (SE 1.0) in UC patients indicating that PST patients improved more than UC patients. However, this difference was not statistically significant (mean difference −0.2, 95% CI −3.7; 3.2).

**Table 3:** Multiply imputed pooled outcomes and costs over 9 months

**Cost category**	**PST (n=88)**	**Usual care (n=87)**	**Difference**
Outcome			
HADS	−3.1 (1.3)	−2.9 (1.0)	−0.2 (−3.7 ; 3.2)
QALY	0.56 (0.02)	0.49 (0.02)	0.03 (−0.02 ; 0.08)*
Cost category			
Direct costs	1751 (221)	2004 (340)	−253 (−1149 ; 476)
Direct healthcare costs	1362 (202)	1829 (326)	−467 (−1340 ; 202)
Direct non-healthcare costs	214 (46)	175 (56)	40 (−117 ; 166)
PST costs	174 (15)	0 (0)	174 (143 ; 206)
Indirect costs	3768 (703)	5889 (1187)	−2121 (−4788 ; 396)
Costs absenteeism	2991 (708)	4319 (951)	−1328 (−3748 ; 823)
Costs presenteeism	778 (251)	1570 (581)	−792 (−2343 ; 209)
Total costs	4795 (671)	6857 (1128)	−2062 (−4698 ; 359)

Presented are means (SEs) and mean differences (95% CIs).

PST = Problem-Solving Treatment; HADS = Hospital Anxiety Depression Scale; QALY = Quality-Adjusted Life-Years.

* corrected for baseline utility.

The mean pooled utility score based on the Dutch tariff for the EQ-5D was 0.71 in the PST group and 0.61 in the UC group. After 3 and 9 months the pooled utility scores were 0.76 and 0.73, respectively, in the PST group and 0.66 and 0.70, respectively, in the UC group. The mean number of QALYs after 9 months was 0.56 (SE 0.02) in the PST group and 0.50 (SE 0.02) in the UC group. After correction for baseline utility, the difference in QALYs between PST and UC was in favour of the PST group (0.03), but statistically non-significant (95% CI −0.02; 0.08).

### Health care utilization

Table 1 presents the health care utilization in the PST and the UC groups at 9 months for patients with complete cost data. In general, health care utilization in the PST group was slightly lower than in the UC group for both general and mental health care.

Sixty-four (73%) PST patients received at least 1 PST session. On average, PST patients received 3 PST sessions. During the study period, 51 (91%) PST patients and 57 (88%) UC patients visited the general practitioner at least once. Thirty-two (36%) PST patients received some form of mental health care (referral to a mental health care provider or prescription of either an antidepressant or benzodiazepine) in comparison with 33 (38%) UC patients during follow-up. Absenteeism in the UC group was substantially higher than in the PST group. There were 2 (4%) patients in the PST group and 4 (6%) patients in the UC group who were absent for more than 180 days.

### Costs

Table 3 presents the imputed pooled mean total costs in the PST and UC group after 9 months. Both direct and indirect costs in the PST group were lower than in the UC group. Indirect costs were the greatest contributor to mean total costs. Mean PST costs amounted to €174 (SE 15). Mean total costs in the PST group were €2062 lower than in the UC group, but this difference was not statistically significant (95% CI -4698; 359).

Total mental health care costs were €485 in the PST group and €259 in the UC group. Although this difference was considerable, it did not reach statistical significance (mean difference €226, 95% CI -3; 448).

### Cost-effectiveness

**Figure 1:**
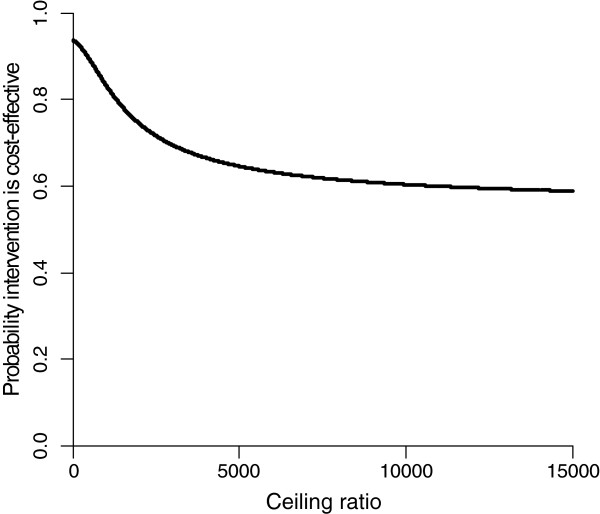
Cost-effectiveness acceptability curve for improvement in total HADS score after 9 months.

The results of the cost-effectiveness and cost-utility analyses are presented in Table 4. The ICER for improvement in total HADS score at 9 months was 8676, meaning that one point improvement extra in the PST group costs €8676 extra in comparison with the UC group. In the CE plane, only 5% of the cost-effect pairs was located in the NE and NW quadrants, while 55% and 40% of the cost-effect pairs was located in the SE and SW quadrant, respectively. The cost-effectiveness acceptability curve in Figure 1 shows that for a ceiling ratio of €0 per point of improvement on the HADS, the probability that PST is cost-effective in comparison with UC is 0.95. For higher ceiling ratios this probability decreases slowly to 0.57.

**Table 4:** Results of cost-effectiveness and cost-utility analyses

**Analysis**	**Sample size**		**Outcome**	**Cost difference (€)**	**Effect difference**	**ICER**	**Distribution CE plane**			
	**PST**	**UC**		**(95% CI)**	**(95%CI)**		**NE**	**SE**	**SW**	**NW**
Multiply imputed analysis	88	87	HADS	−2062 (−4698 ; 359)	−0.2 (−3.7 ; 3.2)	8676	2%	55%	40%	3%
	88	87	QALY*	−2062 (−4698 ; 359)	0.03 (−0.02 ; 0.08)	−65045	4%	88%	7%	1%
Complete cases	56	63	HADS	−2715 (−5858 ; 77)	0.02 (−2.3 ; 2.4)	−113112	1%	48%	49%	2%
	53	59	QALY*	−3085 (−6300 ; -171)	0.04 (−0.01 ; 0.08)	−83380	2%	94%	4%	0%
NHS perspective^†^	88	87	HADS	−253 (−1149 ; 476)	−0.2 (−3.7 ; 3.2)	1065	11%	45%	28%	16%
	88	87	QALY*	−253 (−1149 ; 476)	0.03 (−0.02 ; 0.08)	−7984	23%	69%	4%	4%
Human capital approach	88	87	HADS	−2397 (−5427 ; 393)	−0.2 (−3.7 ; 3.2)	10084	2%	55%	40%	3%
	88	87	QALY*	−2397 (−5427 ; 393)	0.03 (−0.02 ; 0.08)	−75597	4%	88%	7%	1%
Training costs PST excluded	88	87	HADS	−2035 (5669 ; 1259)	−0.2 (−3.7 ; 3.2)	8564	6%	50%	38%	6%
	88	87	QALY*	−2035 (5669 ; 1259)	0.03 (−0.02 ; 0.08)	−64198	11%	81%	7%	1%
Per protocol analysis	41	87	HADS	−3220 (−5744 ; -725)	1.5 (−1.5 ; 4.4)	−2943	0%	16%	84%	0%
	41	87	QALY*	−3220 (−5744 ; -725)	0.03 (−0.03 ; 0.09)	−137279	0%	90%	10%	0%
Observed outliers excluded	88	84	HADS	−1090 (−3492 ; 1175)	−0.3 (−3.8 ; 3.3)	4297	7%	49%	33%	11%
	88	84	QALY*	−1090 (−3492 ; 1175)	0.03 (−0.02 ; 0.08)	−34498	15%	77%	5%	3%

HADS = Hospital Anxiety and Depression Scale; QALY = Quality-Adjusted Life-Year; NHS = National Health Service.

* adjusted for baseline utility score.

^†^ only direct costs included.

**Figure 2:**
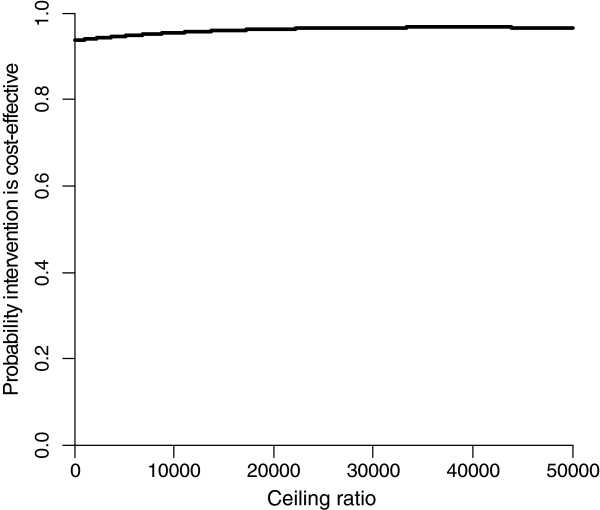
Cost-effectiveness acceptability curve for QALYs after 9 months.

The difference in QALYs after 9 months between the PST and UC groups was very small leading to a very large ICER. The CE plane showed that 88% and 7% of the cost-effect pairs was located in the SE and SW quadrants, respectively. The cost-effectiveness acceptability curve in Figure 2 shows that PST is considered cost-effective in comparison with UC for all ceiling ratios.

### Sensitivity analyses

Results of the sensitivity analyses are presented in Table 4. PST was considered cost-effective in comparison with UC in all sensitivity analyses. In the complete case analyses, differences in clinical effects were small and not statistically significant. Total costs in the PST group were significantly lower than in the UC group. Therefore, PST was considered cost-effective in comparison with UC in the complete case analyses.

In the sensitivity analysis performed from the NHS perspective, costs in the PST group were €253 lower than in the UC group. This difference was statistically non-significant. CEA curves suggest that the willingness to pay should be high to consider PST cost-effective in comparison with UC (data not shown).

Use of the human capital approach led to higher estimates of indirect and total costs in both groups. However, the difference in total costs changed from -€2062 to -€2397 which was not statistically significant. In this analysis, PST was also considered cost-effective in comparison with UC.

Excluding training costs from the PST costs, resulted in lower costs for the PST treatment (€151). The difference in total costs between PST and UC in this sensitivity analysis was -€2035, but this was not statistically significant (95% CI -5669; 1259). The conclusion regarding cost-effectiveness did not change in this analysis.

In the per protocol analysis, the difference in total costs was -€-3220 which was statistically significant (95% CI -5744; -725). The differences in improvement in HADS score and QALY were somewhat larger than in the main analysis, but these differences were not statistically significant. PST was considered cost-effective in comparison with UC.

## Discussion

Our results show that there were no significant differences in effects between PST and UC patients in this population of primary care patients with relatively mild levels of distress. The cost difference between PST and UC was substantial, but not statistically significant. Cost-effectiveness planes and acceptability curves show that PST was cost-effective in comparison with UC. Indirect costs in the PST group were substantially lower than in the UC group and were the greatest contributor to the difference in total costs. However, this difference was mainly caused by 3 outliers in the UC group. Sensitivity analyses confirmed the small differences in effects and that costs in the PST group were substantially lower than in the UC group. Based on these analyses, PST was also considered cost-effective in comparison with UC in this study. Analyses from the NHS perspective suggested that PST is not considered cost-effective in comparison with UC.

UC patients had significantly lower utility scores at baseline than PST patients. Therefore, we suspect that morbidity rates in the UC group were higher than in the PST group. This may also explain the higher costs in the UC group. Additional analyses showed that the differences in direct and indirect costs were only partially explained by the difference in utility score at baseline (data not shown).

One of the strengths of this trial is that it was a pragmatic trial, meaning that we tried to resemble daily clinical practice as much as possible. By applying as few restrictions as possible on patient selection, we think we succeeded in recruiting a population that is representative of the patients with mental health problems seen by the GP. Also, we tried to model the GP’s normal care process as much as possible. Therefore, the results of this study are likely to be generalisable to the rest of The Netherlands and other countries with similar health care systems.

Research suggests that a considerable part of lost productivity costs in mental disorders is caused by presenteeism [36,37]. Therefore, another important strength of this study is that both costs of absenteeism and presenteeism (being present at work, but at reduced work performance) were included, whereas earlier studies included only costs of absenteeism [15,16].

Our study also has some limitations. First, our study was underpowered to detect relevant cost differences which is reflected in the wide confidence intervals around the cost differences. This is a common problem in economic evaluations alongside clinical trials. Because of the heavily skewed distribution of cost data, very large numbers of patients are needed to detect relevant cost differences [38]. Second, the number of patients that did not return all cost questionnaires was considerable (PST group 36%, UC group 25%). However, there were no significant differences between patients with and without complete cost data, reducing the chance of bias caused by selective drop-out. Moreover, both the results of the imputed analysis and the complete case analysis showed that PST is cost-effective in comparison with UC. Finally, the follow-up period of the trial may have been too short. If PST indeed is beneficial in comparison with usual care, then it is reasonable to assume that these benefits extend over many years.

Our results can be compared with two other studies that included an economic evaluation of PST for primary care patients with mental health problems [15,16]. Our results are consistent with their findings that PST is not effective in comparison with usual GP care. These studies showed that PST was associated with higher costs than usual GP care, while in our study lower costs were found in the PST group. Possible explanations for this discrepancy include the length of the follow-up (9 months in this study versus 6 months in the other studies), the higher costs of the PST in the other studies, and the cost categories that were included.

The accompanying clinical trial showed in a post-hoc analysis that a sub-group of more severely depressed patients could benefit from PST [18]. This is in line with earlier research [13,14]. A recent review showed that PST is more effective in comparison with GP care for depression, and mixed anxiety and depression [39]. Thus, it is reasonable to assume that PST should be reserved for patients with more severe mental health symptoms. A potential fruitful avenue is the development of stepped care approaches in which PST is offered to patients who do not recover or deteriorate during a period of ‘watchful waiting’.

## Conclusions

In conclusion, this study shows that PST did not result in improved clinical outcomes in comparison with UC, but did result in substantially lower costs which are explained by the lower indirect costs in the PST group. Based on these results, PST was considered cost-effective in comparison with usual care, although not from an NHS perspective. Since, most of the difference in costs in our study was caused by 3 outliers with very high lost productivity costs, a too strong conclusion on the cost-effectiveness of PST cannot be drawn based on the results of this study.

## Competing interests

The authors declare that they have no competing interests.

## Authors’ contributions

PvO and HWJvM developed the design of the randomized clinical trial and participated in writing the article. BS collected the data. JEB analyzed the data and drafted the manuscript. All authors made substantial contributions to the interpretation of the data, were involved in drafting the manuscript, and gave final approval of the version to be published. All authors read and approved the final manuscript.

## Pre-publication history

The pre-publication history for this paper can be accessed here:

http://www.biomedcentral.com/1471-2296/13/98/prepub

## Author details

^1^Department of Health Sciences and EMGO Institute for Health and Care Research, Faculty of Earth and Life Sciences, VU University Amsterdam, Amsterdam, The Netherlands. ^2^Department of General Practice and EMGO Institute for Health and Care Research, VU University Medical Center, Amsterdam, The Netherlands. ^3^Department of Psychiatry and EMGO Institute for Health and Care Research, VU University Medical Center, Amsterdam, The Netherlands. ^4^Department of Epidemiology & Biostatistics and EMGO Institute for Health and Care Research, VU University Medical Center, Amsterdam, The Netherlands.
